# Climate Change and Professional Responsibility: A Declaration of Helsinki for Engineers

**DOI:** 10.1007/s11948-017-9884-4

**Published:** 2017-03-09

**Authors:** Rob Lawlor, Helen Morley

**Affiliations:** Inter-Disciplinary Ethics Applied, University of Leeds, Leeds, UK

**Keywords:** Climate change, Global warming, Professional responsibility, Responsible leadership, Declaration of Helsinki, Revoke chartered status, Codes of ethics

## Abstract

In this paper, we argue that the professional engineering institutions ought to develop a Declaration of Climate Action. Climate change is a serious global problem, and the majority of greenhouse gas emissions come from industries that are enabled by engineers and represented by the engineering professional institutions. If the professional institutions take seriously the claim that a profession should be self-regulating, with codes of ethics that go beyond mere obedience to the law, and if they take their own ethical codes seriously, recognising their responsibility to the public and to future generations (and also recognising a duty of “responsible leadership”), the professional institutions ought to develop a declaration for engineers, addressing climate change. Our argument here is largely inspired by the history of the Declaration of Helsinki. The Declaration of Helsinki was created by the medical profession for the profession, and it held physicians to a higher standard of ethical conduct than was found in the legal framework of individual countries. Although it was not originally a legal document, the influence of the Declaration can be seen in the fact that it is now enshrined in law in a number of different countries. Thus, we argue that the engineering profession could, and should, play a significant role in the abatement of climate change by making changes within the profession. If the engineering profession sets strict standards for professional engineers, with sanctions for those who refuse to comply, this could have a significant impact in relation to our efforts to develop a coordinated response to climate change.

## Introduction

We will argue that the engineering profession could, and *should*, play a significant role in the abatement of climate change by making changes to the running of their own profession—particularly in relation to self-regulation. We will appeal to the Declaration of Helsinki by the World Medical Association (WMA), arguing that it sets a precedent for the role of a profession in addressing ethical issues. We will argue that, if the engineering profession can coordinate itself in order to set strict standards for professional engineers, and to impose sanctions for any engineers who refuse to follow these new standards (for example, revoking an engineer’s chartered status), there could be real potential to develop a coordinated response to climate change. Ultimately, we will be arguing that the engineering profession should develop a Declaration of Climate Action, comparable to the WMA’s Declaration of Helsinki. We will then explore the idea in more detail by considering, and responding to, a number of objections.

As well as engaging with the academic literature, both in relation to the ethics of climate change and in relation to engineering ethics, this paper also engages directly with the community that the paper is addressing: the engineering profession. A version of this argument was presented at the Royal Academy of Engineering (RAEng) on the 12th of June 2015, at an event which we organised with the RAEng “Engineers and Climate Change: Leadership, Ethics and Responsibilities”,^1^ bringing together academics and engineers from the Academy’s Fellowship.^2^ In addition, a version of the argument was also presented at an event at Engineers Without Borders UK on the 26th of February 2016 (and discussed further in a follow up meeting on the 6th of May 2016). Therefore, as well as engaging with the academic literature, we also respond to objections and concerns from the Fellows of the RAEng, providing philosophical responses to real world discussions that are occurring within the engineering profession. The events were held under the Chatham House Rule, so we do not identify individuals when responding to their comments (unless they have explicitly given us permission to identify them).

We will start by highlighting a tension between the engineering profession’s ethical responsibilities and their actions.

## Words and Actions

The Royal Academy of Engineering (RAEng) and Engineering Council’s joint Statement of Ethical Principles states that professional engineers should have “Respect for Life, Law and the Public Good”, and should “minimise and justify any adverse effect on society or on the natural environment for their own and succeeding generations”. It also recognises a duty of “Responsible Leadership”. (Engineering Council and Royal Academy of Engineering 2014).

Assuming that we recognise that greenhouse gas (GHG) emissions have an impact on the environment, it is clear that a principle that engineers ought to minimise adverse effects on the environment, with explicit mention of succeeding generations, entails a commitment to action to reduce GHG emissions in order to mitigate climate change.

Yet, the majority of carbon emissions come from industries that are enabled by engineers and represented by the engineering professional institutions. Richard Heede emphasises that “nearly two-thirds of historic carbon dioxide and methane emissions can be attributed to 90 entities”. 56 are crude oil and natural gas producers, 37 are coal extractors (including subsidiaries of oil and gas companies), and 7 are cement producers (Heede 2014).

Bill McKibben emphasises the tension between the 565 gigatonnes of carbon that we can afford to emit, if we hope to keep within the 2 °C warming that is considered, by some, to be the limit of safe warming, and the 2795 gigatonnes of carbon already contained in “proven coal and oil and gas reserves of fossil-fuel companies”. (McKibben 2013). There may be some debate about the exact numbers, but the general idea is relatively uncontroversial. For example, Heede states:

The International Energy Agency has concluded that ‘no more than one-third of proven reserves of fossil fuels can be consumed prior to 2050 if the world is to achieve the 2 degree C goal’ (Heede 2014)

Thus, there appears to be a tension between words and actions. The engineering profession recognises their responsibilities to the environment and to future generations, yet engineers have enabled, and continue to enable, these core emitting activities both directly (in relation to the fossil fuel industry) and indirectly (through the creation of industrial and commercial markets for associated goods).

However, it is not the purpose of this paper to consider the extent to which it is the engineers (who *supplied* the energy and goods) who are morally responsible for past emissions (rather than, for example, the consumers who created the *demand*). Our focus is forward looking, focusing on the potential that the engineering profession has to make a positive contribution.

## The Potential of Engineers

We will argue that there are (at least) three reasons why engineers and engineering institutions are particularly well placed to respond to the current climate challenges.

First, as the details above indicate, engineers as a group are particularly well-placed to control and influence the development of key industries responsible for emissions. Changes in engineering practice, therefore, have significant potential to change our current emissions profile.

The second factor that places the engineering profession so well is that it is a profession. As many people have emphasised, climate change involves a collective action problem which is likely to require a co-ordinated response.^3^ The defining characteristics of a profession include (1) a duty to the public and (2) self-regulation. If a profession is self-regulating, it should have both the ability and the necessary mechanisms to orchestrate a coordinated response to collective action problems. And, recognising a duty to the public, professional institutions are underpinned by an awareness that their members ought to act in the public interest, with a focus that extends beyond the individual client or company for which they are currently working. For example, we have already mentioned the RAEng’s and the Engineering Council’s commitment to future generations and to responsible leadership. Thus public interest mandate, when *combined* with our first point above, suggests that the profession *ought* to be under a greater duty than businesses or general members of the public to respond to the problem of climate change, not merely by developing new technologies, but also by regulating the core activities that are contributing to the problem.

The third reason is that professional institutions have a significant degree of deserved public voice. Their pronouncements on issues of importance in their field can be expected to carry weight in wider social discourses as a result of their relevant expertise. Much of the existing discourse considers a future role for various types of emerging and adaptive technologies in both alleviating and mitigating the effects of climate change. In this context, if the engineering profession makes a bold statement about the need to address climate change, and about the profession’s intention to assert their set strict standards, this is likely to be more effective than similar advice coming from other groups.

Throughout this paper, we base our arguments on a certain conception of what it is to be a profession, the core of which is widely shared. Typically, the characterisation of something as a profession is thought to include a role of service to others, self-regulation, making use of a special competence to address a matter of public concern, and having a code of conduct for those within the profession (Cruess et al. 2004; Kipnis 1983; Robinson 2007; Bennion 1969; Davis 1991). We are, of course, influenced by this established literature on the nature of a profession and the responsibility of professionals. However, the current literature focuses primarily on the ethical duties, responsibilities and challenges that face *individual* professionals, and this is the most significant way in which this paper differs from the majority of the academic literature on professional ethics. Our paper focuses not on individual engineers, but on the engineering institutions themselves.

Daniel Wueste notes “A profession is more than a collection of persons with similar expertise and jurisdiction. It is a social institution”. (Wueste 1994, p. 17). Similarly, Dennis Thompson states that “ethics, both as an academic discipline and as concrete practice, has tended to focus either on relations among individuals, or on the structures of society as a whole. It has neglected that middle range of intermediate associations, of which institutions are the most durable and influential. Institutions are the site of many of our most difficult moral problems, as well as the source for many of our most promising solutions”. (Thompson 2007). In this paper, we argue that the engineering professional institutions have the potential to be the source of a very promising response to climate change.^4^

## A Declaration of Helsinki for Engineers

Our suggestion in this paper is that the engineering professional institutions should take advantage of their potential to provide a co-ordinated response to climate change. If the engineering profession can coordinate itself in order to set strict standards for professional engineers, with sanctions for those who refuse to comply, this could have a significant impact in relation to our efforts to develop a coordinated response to climate change.

Regarding sanctions, we suggest that the options currently used by professional institutions are sufficient. If an engineer is found guilty of professional misconduct, she could receive an official warning, or she could be fined or—in more serious cases—could have her chartered status suspended or even revoked. In addition, details of the wrongdoing are often published within the profession.

In order to see clearly the potential that professional institutions have, we should consider what has been achieved by professional institutions in the past. Arguably the most notable example, which we will focus on here, is The World Medical Association’s Declaration of Helsinki, first developed in 1964, reforming practices in medical research, addressing ethical issues relating to medical research on human participants (World Medical Association 2008).

The Declaration of Helsinki was not originally a legal document. Rather, it was created by the profession for the profession, and it held physicians to a higher standard of ethical conduct than was found in the legal framework of individual countries. However, although it was not *originally* a legal document, the influence of the Declaration can be seen in the fact that it is now enshrined in law in a number of different countries.^5^

Our suggestion is that engineers should aim to produce a similar document, which is similarly created by the profession, for the profession, aiming to set a higher standard of ethical conduct than is currently found in the law—with the aim of reducing carbon emissions significantly.

If the professional institutions take seriously the claim that a profession should be self-regulating, with codes of ethics that go beyond mere obedience to the law, and if they take their own ethical codes seriously, recognising their responsibility to the public, and to future generations, they ought to take our suggestion very seriously. They need, we suggest, to develop a Declaration of Helsinki for engineers—a declaration of climate action, or more broadly, a declaration of global responsibilities.

## Similarities and Differences

We acknowledge that there are important differences between the medical profession and the engineering profession, and between the issue of climate change and the issue of research ethics. Before acknowledging the differences, however, it is worth noting some key similarities between engineering and medical research.^6^ Both have the potential to make significant contributions to human well-being. Both also have potential to do serious harm to individuals or groups. Furthermore, these two points are not isolated issues: there is potential to harm some in the process of seeking benefits for others. As a result, there is a temptation to impose risks on some, arguing that the ends justify the means.

To a large extent, the logic of the “ends justify the means” was exactly what the Declaration of Helsinki was designed to counter. The Declaration emphasises that:

Some research populations are particularly vulnerable and need special protection. These include those who cannot give or refuse consent for themselves… (World Medical Association 2008)

In the context of climate change, the vulnerable populations who cannot give or refuse consent would include future generations, and also individuals and nations with little or no political power. Rejecting the idea that the benefits to others can justify the harms to the vulnerable, the Declaration continues:

Medical research involving a disadvantaged or vulnerable population or community is only justified if the project is responsive to the needs and priorities of this population or community and if there is a reasonable likelihood that this population or community stands to benefit from the results of the research. (World Medical Association 2008)

Nevertheless, it is clear that there is a difference between climate change and medical research, particularly relating to the complexity of climate change,^7^ and challenges relating to economic competition.^8^

First, we will argue that there is one complexity that we do *not* need to be concerned about, because it is misguided. We do not need to worry about the difficulty of not being able to say that this engineer is responsible for *this particular* set of emissions, or for *this particular* harm. There is no reason why this difficulty should make it impossible for us to hold engineers *morally* responsible for their actions. If we can provide a set of regulations which—if adhered to—would help to minimise the risk of harm, this is all we need. Once we have these regulations, we can criticise and punish those who refuse to comply. This is no different from any other regulations regarding—for example—pollution. There is no need to connect any particular harm to any particular act. The regulations are justified by the benefits that they can bring. Once the regulations have been put in place, the punishment that any particular engineer receives needn’t be understood as a punishment for any particular harm that they are *causally* responsible for. It is perfectly justifiable if the punishment is simply punishment for an engineer’s *non*-*compliance* with rules which—if adhered to—would help to combat climate change.

The difficulty that we take more seriously, and which we recognise as a very real challenge, is the difficulty in setting out what those regulations should be. In this paper, we will not give a detailed account of what we think should go into a declaration of climate action. We will, however, highlight two general approaches that are available (and which are not mutually exclusive).

On the one hand, we could set out a list of specific prohibitions and requirements. On the other hand, we could include principles that are deliberately vague, and require interpretation. The latter option may not sound promising. Some might suggest that this would be no different from the principles that we already have—which suggest that engineers should “minimise and justify any adverse effect on society or on the natural environment for their own and succeeding generations” (Engineering Council and Royal Academy of Engineering 2014)—but which currently do not seem to be effective.

Before we dismiss this second approach, however, there are three important points to emphasise. First, there are good reasons to include vaguer principles that allow for interpretation. Consider this in relation to risk, negligence and recklessness. It is simply not possible to predict every way in which it is possible for a person to act—for example—recklessly. If we relied only on the first type of principle, we would be very prone to wrong-doers finding loopholes, arguing—for example—that there was no law against driving while blind-folded.

This brings us to the second point to emphasise. These vague rules are already used effectively both legally and by professional institutions, in relation to (for example) risk and regulation. With the exception of Alabama, most states do not have a law stating explicitly that it is illegal to drive a car while blindfolded (Atlas 2012). It does not follow, however, that one could drive a car while blindfolded and expect not to be prosecuted. The driver would be prosecuted for a more general crime, such as “reckless driving”. But what counts as reckless is, of course, vague, and open to interpretation. But that is the point. It is this vagueness that explains why most states do not feel that they have to follow Alabama’s lead, having a law explicitly outlawing driving while blindfolded.

Similarly, the law on negligence works in the same way. We cannot list every way in which a person could be negligent. In law, negligence requires “conduct that falls short of the standard expected of a person where a duty of care is owed”. (Cruz 2013, p. 233). To a large extent, this is vague. In the context of medical law, a defence against a charge of negligence would typically take the form of the individual arguing that his or her actions were consistent with what would be expected of a doctor. For example, it could involve evidence from other doctors stating that they would have acted in the same way. On the face of it, this looks like a problem in relation to our argument, relating to climate change. However, this is only a problem if we think that professional standards cannot change. A profession, for example, can come out and say that existing standards are not acceptable. Plausibly, this is the best way to understand the Declaration of Helsinki. This was an example of the medical profession making a point of stating explicitly that the standards of the time were not appropriate, and needed to be changed. Similarly, the engineering profession could come out and say that engineers are taking too many risks, or—in the context of climate change—engineers are not doing enough to protect future generations.

Now though we need to find a way to make it clear that we are changing the standards—and this is where we find limits to the appeal to vague rules that leave room for interpretation. This brings us to our third point. The two options are not mutually exclusive—and what is more neither are they independent of each other. Consider driving laws again. We can have a specific law, stating that one must not drink and drive (even stating the specific alcohol limits in terms of blood alcohol concentration), while also having a vague rule against reckless driving. And here the interplay is relevant: if people understand that it is illegal to drive while drunk, it is reasonable to expect them to realise that it is also similarly reckless to do other things that are at least as dangerous as driving drunk. As such, the clear, specific laws can be helpful in the interpretation of the more vague laws that leave room for interpretation. They can help to clarify what standards are appropriate.

We suggest, therefore, that a declaration of climate action should allow for both. The difficult question is, what specific laws should it include? This is not a question that we intend to answer in this paper. We will, however, offer a couple of suggestions—to kickstart the debate. Given McKibben’s point about the conflict between the amount of fossil fuel we have, and the amount that can be burnt, one could make a case for a prohibition on *new* explorations. Engineers should not be permitted, professionally, to work on *new* coal mines or new oil drilling. Given that gas is quite considerably better than coal, but still worse than renewable energy sources, there would be an argument to be had about whether this prohibition would extend to fracking or not.

Similarly, we might consider introducing restrictions on when concrete can be used (when there are other viable alternatives). In some cases, the solution might involve giving teeth to existing guidelines or existing certification schemes. For example, the Forest Stewardship Council (FSC) set standards for logging, and certifies products that meet their standards. However, these standards are not legal requirements, and the FSC standards are typically higher than the standards that are legally required. Therefore, professional institutions could give teeth to the FSC certification by insisting that engineers should refuse to work using timber that is not FSC certified. Similarly, one engineer^9^ suggested the Building Research Establishment (BRE)’s Building Research Establishment Environmental Assessment Methodology (BREEAM). (Building Research Establishment n.d.)

We are not committed to these particular suggestions. Our aim is simply to give an idea of the *sort* of approach that could be used. An appeal to the standards that would be set by BREEAM, for example, for those working in construction, would be one way in which a professional institution could make a clear statement about what should be considered appropriate standards for those working in relevant areas. The explicit rules and guidelines would also then feed into the vague regulations that require interpretation in a way that is similar to our example above in which drinking and driving laws and feed into the more general reckless driving). This explicit restatement of standards would then allow the professional institutions to hold individual engineers accountable, and to claim that they were not doing enough to minimise adverse effects on future generations.

## Further Details: Objections and Replies

Of course, we are well aware that this idea may be considered controversial and that many will respond with objections and challenges. In this section we will reply to a number of objections, and also use these replies to provide a more detailed account of what we are arguing for.

### Not an Original Solution

One response that could be made to our proposal is that it is too obvious to be uninteresting: there is nothing significant or noteworthy about suggesting that institutions should address ethical issues by having a code of ethics, and this may sometimes make demands that go beyond doing what the law requires.^10^ Indeed, the similarity to current codes of ethics, whilst supporting our argument, may mislead one into thinking that there is nothing new here.

We believe that this objection is misguided: this is a radical and original suggestion that is not currently being discussed. To see this clearly, consider the following question: what is the typical focus of discussions about how to resolve climate change? To a large extent the contrast is usually between action taken at the individual level versus action taken by governments, considering to what we should focus on each.^11^ The general consensus (to the extent that there is one) is that a political solution, involving regulation or a carbon tax, would be *required* to make the necessary reduction in emissions achievable, and that the key focus therefore should be on government action.

In this context, our paper is interesting in two distinct ways.

First, appealing to the example of the Declaration of Helsinki, we argue that professional institutions can adopt a leadership role and play a significant part in making this political action a reality.

Second, and even more radically, we suggest that it may not be true that political action, introducing legislation and laws, is required in order to mitigate climate change. Given the reliance on engineers, a strong and coordinated action by the engineering profession could itself make a significant difference in how we respond to climate change. Thus, we are aiming to cast doubt on the mainstream view that only political action by national governments could be sufficient to achieve the kind of coordinated response that is required. We are suggesting that there might be another alternative, with the engineering profession at the forefront.

In addition, we are also making a more significant statement in relation to the responsibility of engineers. Of course, many people talk about engineers’ responsibilities, suggesting that engineers ought to think about the environment when making design choices. But what needs to be recognised here is that we are suggesting something *much* more radical than this. We are asserting that climate change is fundamentally an issue for the engineering profession in the same way that we now recognise that research ethics is fundamentally an issue for the medical profession, such that the engineering profession *ought* to play a *leading* role in addressing problems relating to climate change. We are suggesting that it is plausible to think that the engineering profession could be the institution that could take the lead in achieving the large scale coordinated action that could be a significant advancement in the effort to reduce global emissions such that we can limit further climate change. As far as we know, no one else is suggesting anything as radical as this.

Far from being obvious and uninteresting, the more reasonable objection would be that our suggestion is too radical and far-fetched. Except again, this is where the Declaration of Helsinki becomes a powerful part of our argument, because the lessons we learn from the history of the Declaration of Helsinki is that such a declaration from a profession really can be a powerful tool, such that it may not be unreasonable to suggest that engineers could lead the way, and (if they choose to) could become the most important players in tackling climate change. Engineers could (as a profession) refuse to enable certain activities that are harming the planet, and the engineering institutions is in a position to *coordinate* this solution. Thus, they could play a pivotal leadership role in reducing emission, and in influencing future law.

What we are suggesting here, in terms of the *extent* to which the engineering profession would be *taking the lead* in responding to a global challenge, would be unprecedented in the engineering community. It is for this reason that we use an example from medicine—the Declaration of Helsinki—rather than an example from engineering, because there is no previous engineering equivalent to appeal to.

### Anthropogenic Climate Change is Not Really a Problem

This is not the place to provide a thorough and detailed account of the scientific evidence. However, a couple of points are worth mentioning. First, there is overwhelming scientific consensus (Oreskes 2004; Cook et al. 2013; IPCC 2014a, b).

In addition, the evidence is *not* based solely on observations of correlation. It is also based on sophisticated climate models, which—crucially—can be *tested*. That is, we can put in data from earlier times in order to test how accurate their predictions are by comparing the results that are predicted by a model, based on the inputted data, with the actual results observed in the real world. In this way, models can be refined until they consistently make accurate predictions such that we then have good reason to trust their predictions for the future. With “very high confidence”, the summary of the IPCC Fifth Assessment Report (WGI AR5) states

Climate models have improved since the AR4. Models reproduce observed continental scale surface temperature patterns and trends over many decades, including the more rapid warming since the mid-20th century and the cooling immediately following large volcanic eruptions (very high confidence). (IPCC 2014b)

The IPCC Fifth Assessment Report also reports on the likely impacts of climate change, stating:

Continued emission of greenhouse gases will cause further warming and long-lasting changes in all components of the climate system, increasing the likelihood of severe, pervasive and irreversible impacts for people and ecosystems. (IPCC 2014a, p. 8)

Impacts highlighted by the report include the following:

Throughout the 21st century, climate change is expected to lead to increases in ill-health in many regions and especially in developing countries with low income, as compared to a baseline without climate change (high confidence). Examples include greater likelihood of injury, disease, and death due to more intense heat waves and fires (very high confidence); increased likelihood of under-nutrition resulting from diminished food production in poor regions (high confidence); risks from lost work capacity and reduced labor productivity in vulnerable populations; and increased risks from food- and water-borne diseases (very high confidence) and vector-borne diseases (medium confidence). (IPCC 2015, pp. 19–20)

In addition,

Throughout the 21st century, climate-change impacts are projected to slow down economic growth, make poverty reduction more difficult, further erode food security, and prolong existing and create new poverty traps, the latter particularly in urban areas and emerging hotspots of hunger (medium confidence). Climate-change impacts are expected to exacerbate poverty in most developing countries and create new poverty pockets in countries with increasing inequality, in both developed and developing countries. (IPCC 2015, p. 20)

And:

Climate change over the 21st century is projected to increase displacement of people (medium evidence, high agreement). (IPCC 2015, p. 20)

And:

Climate change can indirectly increase risks of violent conflicts in the form of civil war and inter-group violence by amplifying well-documented drivers of these conflicts such as poverty and economic shocks (medium confidence). Multiple lines of evidence relate climate variability to these forms of conflict. (IPCC 2015, p. 20)

Given the strength of the evidence to support the claim that anthropogenic climate change is a reality, and given the significance of its expected impact, the evidence clearly supports our claim that it is vitally important that the engineering professional institutions make a clear and explicit declaration about the need to reduce GHG emissions, and to make it clear that this is necessary even if (or especially if) this puts professional engineers in conflict with their employers.^12^

The impacts listed above represent a significant risk to human wellbeing. When one also considers that the Engineering Council and Royal Academy of Engineering’s shared Statement of Ethical Principles requires engineers to “minimise and justify any adverse effect on society or on the natural environment for their own and succeeding generations” and to “hold paramount the health and safety of others” it is clear that climate change represents an area of legitimate and significant ethical concern for the engineering profession.^13^

### The Applicability Across Engineering Domains

Following from the previous section, we also acknowledge that there may be another objection, even if we do recognise that climate change is an important issue and is relevant to *some* engineers. The objection we have in mind could come in two ways. One would be look at the activities, professional responsibilities and fields of influence of engineers who are not working in the fossil fuel industry, or in other areas (such as aviation) that are notorious for their GHG emissions. Consider, for example, an audio engineer or biomedical engineer. The objection is that a declaration that covers all engineering domains will be too broad. It is just not plausible to think we need a declaration of climate action for audio engineers.

The second version of the objection focuses on our comparison with the Declaration of Helsinki and the medical profession. This version of the objection states that the Declaration of Helsinki is not an appropriate model, because the relationship between research ethics and medics is not analogous to that of climate change to engineers.^14^

Addressing the second version of the objection first, it is important to recognise that the difference between the two cases may not be as significant as some may think. In relation to medicine, different doctors follow different paths. Some focus on teaching, others will focus on management, and some will focus on research. Some might focus on more than one of these areas, but that would be fairly unusual, and some may not get involved in any of these areas, focusing solely on the treatment of patients. Those who did get involved in research would be required to undertake good clinical practice (GCP) training—which is largely based around the Declaration of Helsinki. But many doctors would not need to do this, precisely because they are not involved in research.^15^

In relation to engineering, while it may be true that the impacts on climate change are *less* significant for some engineers’ work than for others, it is not clear that this means that we should assume that a declaration of climate action would be irrelevant or of no great significance for audio engineers, for example. While there are clearly areas of engineering that have a greater impact on climate change than others, as highlighted by Heede, we should not lose sight of the fact that climate change is ultimately the result of the *accumulation* of emissions from a range of different sources. While it is true that concerns about emissions may not be significant for all engineers, neither is it an issue that should be thought to be relevant only to a small fraction of engineers. The average emissions for a person, per year, differ according to where the individual lives. Mike Berners-Lee estimates that the “consumption footprint” (in tonnes of CO_2_e^16^ per year) is 15 tonnes for the average UK inhabitant and 28 tonnes for the average North American, compared to the world average of 7 tonnes (Berners-Lee 2010, p. 139).

Much of this difference is likely to be due to the impact of particular consumables (such as meat, cars and flights), but it is far from clear that these are the only consumables that we need to care about. In “Delaying Obsolescence” Rob Lawlor focuses on the problems associated with everyday consumables like computers, phones and cameras, and—focusing on the issue of obsolescence (and of upgrading these consumables increasingly frequently)—he considers the ways in which different design decisions and different approaches to business and marketing can mitigate or exacerbate the problem (Lawlor 2015). Ultimately, he argues that the design of consumables *is* something engineers have a responsibility to think about, in the context of climate change. While he doesn’t list particular figures relating to the home entertainment systems that the audio engineer might work on, he does quote Giles Slade emphasising that in 2003 “63 million working PCs dumped into American landfills” and in 2004 this increased to “315 million”. (Slade 2006, p. 1). To emphasise the impact of replacing these products earlier rather than later, Lawlor quotes Berners-Lee’s figures for the CO_2_e emissions for computers: “720 kg CO_2_e a 2010 21.5-inch iMac… Even before you turn it on, a new iMac has the same footprint as flying from Glasgow to Madrid and back”. (Berners-Lee 2010, pp. 124–125).

Our reason for including the above discussion is that we are conscious that it is not always obvious to engineers that their work has climatic impact. Nonetheless, we would not want to push that argument too far, and we certainly accept the general point that the declaration of climate action will be more significant in relation to civil engineers working on large building projects, or petroleum engineers working on the extraction of fossil fuels, or mechanical engineers working on the design of new cars. But just because it will be *more* significant for these engineers, it does not follow that the declaration would be irrelevant to other engineers.

More importantly though, why would it matter if the declaration of climate action would not apply to all engineers? We have already highlighted the fact that many doctors are not involved in research. Yet, the WMA introduced the Declaration of Helsinki. Similarly, not every citizen of a country drives. That does not mean that governments should not introduce driving laws? Very few people ride motorcycles, but governments still have laws that relate specifically to riding motorcycles—because a government is the relevant institution to introduce a law (even if it only applies to a fraction of citizens). Similarly, if we have provided a compelling case for why the engineering profession is the appropriate institution to regulate the actions of engineers, in order to coordinate action to reduce emissions, this argument is not undermined by pointing out that it may not be relevant to *all* engineers.

Earlier we stated that “the majority of carbon emissions come from industries that are enabled by engineers and represented by the engineering professional institutions”. Even if it was only a small fraction of engineers who worked in these industries, this would not make a significant difference to our argument. If we want to regulate these activities, and if these activities are enabled by engineers, the engineering professional institutions have the potential to make a significant impact and to take on a role of leadership to combat climate change.

Even if you believe it is an oddity that electrical engineers working on phones and chemical engineers working on pharmaceuticals and petroleum engineers working in the fossil fuel industry all co-exist within a single profession, the fact of the matter is that the engineering profession acts, and is named, as a single profession. This gives the profession a strong voice, putting it in an excellent position to take on a significant and influential leadership role.

### Overstepping the Mark

Some worry that professional institutions would be overstepping the mark in imposing significant restrictions on engineers. This, they argue, is the role of the law, not the role of professional institutions. We have two main responses to this. The first is an appeal to precedent. One clear precedent is the example we have already focused on: the Declaration of Helsinki. If we want an engineering precedent, however, consider the case of bribery. Prior to the UK Bribery Act 2010, whether or not one was breaking the law would depend on the law in the country you were working in. However, many professional institutions didn’t simply wait for the Bribery Act 2010. Even before 2010, many institutions explicitly stated that bribery would be considered misconduct and would not be tolerated. In both cases, professional institutions have been willing to impose requirements that go beyond what is required by law.

Our second response is to emphasise the fact that—far from going beyond its proper role—self-regulation is a defining characteristic of a profession. Going beyond the law, and stating ethical standards that go beyond the law, is precisely what a professional institution ought to do. The Declaration of Helsinki is very explicit in emphasising its relation to the law:

No national or international ethical, legal or regulatory requirements should reduce or eliminate any of the protections for research subjects set forth in this Declaration (World Medical Association 2008).

### Constraints on Engineers

Another common objection that fails to appreciate the importance of self-regulation can be put as follows:

We need to take seriously the extent to which engineers were constrained by circumstances – particularly by the fact that, in most cases, engineers will be working for an employer. To a large extent, therefore, engineers are constrained by what their employers are willing to do.

This gets things the wrong way round. On the contrary, employers should be constrained by what professional engineers are willing to do. Otherwise, a professional code of conduct is just for show. If engineers are to be considered professionals, belonging to a self-regulating profession, engineers should be expected to have standards that go beyond obeying the law, and they must be willing to refuse to act in ways that conflict with the profession’s codes of conduct.^17^ A profession that *only* requires its members to act within the law does not look like a profession that is self-regulating in any meaningful sense. This idea is crucial to our argument.

### Draconian

In contrast to the earlier objection that our proposal was not original, and was just the usual appeal to the benefits of a code of ethics another objection is that our proposal is too radical and too strict. One engineer, at an event we organised with the RAEng, called our proposal “draconian”. On the face of it, it may seem to be draconian to suggest that we could revoke someone’s chartered status for doing something that, by current standards, looks innocuous—like everyday engineering.

Again, we suggest that this objection demonstrates a failure to understand the nature of the problem and the nature of the solution. We must recognise that it is the very nature of the problem of climate change that dictates that the correct solution will require people to change their behaviour even in cases where—until recently—those behaviours seemed benign and unproblematic. In many cases, individual actions, would be harmless, if they were done in isolation, and there would be no need for prohibitions. But, when multiplied, these same actions cause significant harm. What is needed, therefore, is an approach that sets *new rules*, and *new standards*, such that people change their behaviour, such that the engineering profession as a whole can act in a coordinated manor to reduce emissions. Judged by old standards, these new rules and standards will look draconian. But to judge these new rules by old standards (not thinking in terms of the impacts of emissions) is inappropriate and suggests a failure to appreciate the nature of the problem.

### Not Strict Enough

In contrast, others suggested that it would not be effective because the threat of punishment would not be sufficient, because having one’s chartered status revoked is not comparable to a doctor being struck off. However, this under-estimates the impact of a threat to revoke chartered status in two ways. First, although it is true that one can work as an engineer without being chartered, there is a difference between choosing not to be chartered, on the one hand, and losing one’s chartered status on the other hand. Having one’s chartered status revoked would be career changing.^18^

Second, we should not under-estimate the impact that this would have indirectly, sending a powerful message not only to chartered engineers, but also to the public and to the government. The historian, Simone Turchetti, commenting on the discussion following our presentation of this paper at the Royal Academy of Engineering, wrote:

I felt that at one point the discussion was revolving too much around the issue of whether or not a ruling (say for instance on membership) could be enforced. I think this is somewhat detracting from what in my view should be an important endorsement for the academy *regardless* of whether rulings should be made. There are plenty of other cases in history (1938, Royal Society on academic refugees from Nazi Germany; 1975 Asilomar conference on genetic engineering) when experts have agreed to mobilize and independently endorse ethical principles as a way to inform practices in the community and not necessarily with a view of ruling what other experts should be doing. If this is what the Academy wants to do in relation to climate change (i.e. set new ethical guidelines), I think the resonance of its decisions could be felt in the engineering community and the wider society.^19^

### Alternative Solutions

Some also argued that this sort of solution, coming from the engineering profession, would not be the best solution available. We should be focusing, instead on a political solution, such as a carbon tax, or a system of licensing for businesses.

Here, it is important to stress that we are not claiming that our engineering-focused solution is the only solution available, or even the best solution available. Clearly, there is a lot that could be done at the political level. We simply claim that—so long as the political solutions are not forthcoming, or are not proving sufficient—there is something that the engineering profession can do to have a positive impact, and that it is something that the professional institutions ought to be doing, given the duties that the profession has, in relation to the public good and responsible leadership.

### National Disadvantage

Finally, another objection drew attention to the global nature of the problem. The concern here is that if professional institutions in one country—say, the UK—implements something like this unilaterally, while others carry on as usual, we will simply put engineers chartered by UK at a disadvantage, and the work will simply be done by the engineers, and companies, of other countries.

This is clearly a legitimate concern, and one that may impose pragmatic limits on what should be done. However, there is a danger that this objection can be overstated, and used to block any meaningful action. (Of course, it should be obvious that this objection does not only apply to the profession imposing restrictions on engineers. The same argument applies to governments looking for political solutions.)

While this complication cannot simply be ignored, there is good reason to think that it is a mistake to think that the only options are to get unanimous political agreement or to give up, and do nothing.

The exact same argument, appealing to national interests, were used to object to regulations addressing bribery and corruption. Writing about the Church Committee in America in the 1970s, Frank Vogl wrote:

At the opening hearing [Douglas] Haughton [of Lockheed] challenged Senator Church by declaring that bribery was part of global business. It was the way things got done. He admitted that his firm had made foreign payments to politicians in the previous four and a half years of around $22 million. Republican Senator John Tower of Texas added that it was unfair that American companies stop making bribes if foreign rivals engage in such practices. (Vogl 2012, p. 165)

And:

The arguments that were being made in the Church hearings by both leading Republicans and Democrats would be repeated over the next two decades in many countries as governments struggled to determine whether foreign bribe paying by their companies should be criminalized or whether bribes should just be accepted as part of the global way of doing business. (Vogl 2012, p. 166)

Similarly, in relation to the UK, he wrote:

In the mid-1990s, former UK trade minister Lord Young told me in a radio discussion on corruption that British firms had to compete around the world to ensure that good jobs were maintained in the UK. To compete, he suggested, might mean doing what everyone else did and pay bribes. (Vogl 2012, p. 181)

Nevertheless, the US Foreign Corrupt Practices Act was introduced in 1977, and the UK’s Bribery Act was introduced in 2010. Similarly, focusing on the engineering profession, after acknowledging the 2010 Bribery Act, the Institution of Civil Engineers’ Advice on Ethical Conduct states:

Until recently, it has been a standard justification for such behaviour that competitors indulge in these practices, and that failure to do so may disadvantage companies who are not dishonest. This can never be accepted as an excuse for members of the ICE to participate in bribery and corruption. (Institution of Civil Engineers 2012)

As such, it is clear that—like the Declaration of Helsinki—this Advice on Ethical Conduct explicitly demands standards that are (sometimes) stricter than legal requirements. Furthermore, it is not considered controversial or draconian to find an engineer guilty of professional misconduct, and even to revoke chartered status, if an engineer is found guilty of corruption or bribery.

Thinking back to the point we made above about the indirect impact a declaration of climate action might have, by sending an important message, this also needs to be considered in the context of professional institutions in a particular country taking the lead in regulating the activities of engineers, Mike Berners-Lee and Duncan Clark highlight various indirect effects, and rebound effects, which reduce the (direct) impact of individual nations reducing their emissions (Berners-Lee and Clark 2013, pp. 47–63). Rather than becoming pessimistic, suggesting that nothing that we can do will make a difference, they write:

As for voluntary carbon and energy savings at the personal, corporate and national level, their real value may be less about reducing emissions directly and more about showing leadership and changing cultural norms… (Berners-Lee and Clark 2013, p. 63)

They continue,

…if we accept that our green efforts are as much about creating social and political ripples as they are abut directly stemming the flow of carbon into the atmosphere, that suggests that we may need to think more about maximising those ripples. (Berners-Lee and Clark 2013, p. 63)

Again, this is something that a declaration of climate action could do very successfully. To make the comparison with bribery again, consider the implications of the following quotes from Vogl about the Church Committee in America in the 1970s:

The members of the [Church] committee knew that powerful and consistent publicity about corporate foreign bribery would, over time, help to create a broad sense of public disgust that would translate into support for tough legislation. (Vogl 2012, p. 163)

[Stanley] Sporkin [the director of enforcement at the Securities Exchange Commission] was convinced that getting big firms to stop paying bribes demanded public humiliation and that the public had to be brought gradually to the understanding that the abuse was rampant and unacceptable. (Vogl 2012, p. 164)

While it is true that we must acknowledge the issues about competition and the competitive disadvantage of imposing restrictions on one nation’s engineers, we must also remember that these competitive elements are the very reason that problems of this nature often require regulation to solve the problem. And, of course, we do not typically think that it is regrettable that efforts have been made to challenge corruption. Again, therefore, we urge the engineering institutions to demonstrate responsible leadership in this area.

## Conclusion

We have argued that the existing ethical principles, already recognised by the engineering profession, obligate engineers to act in response to the serious challenge of climate change. There is a sense in which we are simply restating the principles that the profession is already committed to. Given that this paper relies heavily on an appeal to consistency, this should be expected. However, given that we have more fossil fuels than we can burn but yet exploration for more fossil fuels continues, and given that engineers are at the heart of the fossil fuel industry, enabling the core emitting activities, and given that we are not succeeding in reducing our emissions, it is clear that the current codes and statements of ethical principles are not sufficient.^20^

These ethical commitments need to be given teeth. It is not enough for a code of ethics to state that engineers should think about future generations. This will not make a difference to engineering practice unless we ensure that engineers know that the choices they make could result in sanctions, such as the revoking of chartered status. Furthermore, if the emissions relating to engineering activity are to be reduced, we will need new standards. Presumably, this is where the charge of being Draconian comes from. Activities and practices that are assumed to be acceptable, and which would not currently lead to accusations of unprofessional conduct, will have to be re-assessed, and some will need to be judged to be unacceptable.

If this is to be fair though, and if engineers are to be given fore-warning such that they can change their practices rather than being caught by surprise and punished for something they believed to be standard practice, the professional institutions will have to make a clear declaration of their intent. They will need to make it clear that they are implementing new regulations, imposing new standards on engineers, with the intention of making a real change to engineering practice, in order to reduce emissions from engineers within their institution, as well as sending a powerful message to other professional institutions, and to the public and to governments, fulfilling their obligation of responsible leadership.

These are the reasons why we suggest that professional institutions in engineering ought to develop a declaration of climate action, despite the fact that we have argued that—in some sense—engineers should *already* recognise the duty to address climate changed, based on existing ethical principles that the professional institutions already endorse.

## Footnotes

1 A summary of the event, from Dr. Andrew Chilvers (Policy Advisor at the RAEng), can be found here (Chilvers 2015). Also see (IDEA 2016) for more about the event.

2 See Engineering Council and Royal Academy of Engineering (2014).

3 For an overview, see Garvey (2011, pp. 61–66). Although it doesn’t focus on climate change, much of this debate is influenced by Hardin (1968). For discussions of similar issues with a more specific focus on engineers, see Davis (1991) and (Lawlor 2016) and (Lawlor and Morley 2016).

4 It is worth noting, that Stephanie Collins and Holly Lawford-Smith have argued that there can be a duty to *collectivise*—that is, for individuals to work to form institutions in order to make collective action possible, if the necessary institutions don’t already exist. (Or, in some cases, if a collective already exists, there may be a duty to reform or re-organise an institution in order to be more effective.) (Collins 2013; Collins and Lawford-Smith 2016; Lawford-Smith 2015). In this context, it is worth emphasising the significance of the fact that the professional institutions have a head start here. The relevant institutions already exist – and already have a significant role in society. Our argument, in this paper, is that people in these institutions need to recognise the potential of their institutions and to work to get to the point where the professional institutions are able to make a significant contribution.

5 For example, in the UK, Finland, Australia, China, Brazil and Belgium. We are grateful to Josie Freear for providing information about the countries which have incorporated the Declaration of Helsinki into law. (Josie Freear was a research assistant on Rob Lawlor’s AHRC project, “Climate Change, Ethics and Responsibility: an interdisciplinary approach”.)

6 Here, we acknowledge that Schinzinger and Martin also make a similar comparison, characterising engineering as social experimentation (Schinzinger and Martin 2000). However, we would also emphasise the fact that their focus is somewhat different from ours. While both they and we compare engineering with medical research, we do so in different ways with a different focus. They focus on uncertainty, and the idea that engineers do things with the intention of benefitting people, without fully knowing what the consequences are going to be. They focus on the significance of (1) acting in partial ignorance, (2) making decisions in the face of uncertainty, and (3) the reliance on knowledge gained about products before and after they leave the factory. In contrast, the comparison that we highlight is based on the fact that, in both cases, there is a temptation to argue that the ends can justify the means. And this is largely what the Declaration of Helsinki was intended to challenge.

7 Of course, we acknowledge that there are complexities, and difficult cases, in medical research too.

8 Which we address later in the paper, in the section on “National Disadvantage”, where we highlight the comparison with bribery, particularly in the way that many individuals challenged the introduction of regulation by arguing that regulation would put companies at a competitive disadvantage.

9 This suggestion was made at the Engineer’s Without Borders event in February 2016.

10 We are grateful to an anonymous reviewer for raising this objection, and for prompting us to add this new section to our paper.

11 For example, see Jamieson (1992, 2007), Sinnott-Armstrong (2005), Gardiner (2014, pp. 307–308), Klein (2015), Sandel (2013), Harford (2012, pp. 154–180), Roodhouse (2007).

12 In relation to this, it is worth noting that the WMA (and other medical institutions across the world) have taken a leadership role here, issuing statements regarding climate change (World Medical Association 2015; Australian Medical Association 2015; Health and Environment Alliance 2015). Also see the section “Constraints on Engineers”.

13 Another reason for thinking that we do not need to worry too much about changing engineering practice, and ought not to make any significant effort to reduce our emissions, is that there might be a different approach to the problem of climate change: for example, geoengineering. We do not intend to address this possibility in detail here, and we do not even dismiss this possibility. We simply make one point: if geoengineering does prove to be the solution, then—at that point—the engineering profession can declare the Declaration on Climate Action to be unnecessary, and no longer in effect, and we can relax or abolish the various regulations. But if some suggest that we should just carry on using fossil fuels on the *assumption* that geoengineering will provide the solution, we suggest that the burden is on them to prove that the *probability* of this being a viable solution is high enough to be consistent with the engineering profession’s obligations to public health and safety, including future generations. The burden is not on us to prove that this is *not* possible. For the moment, the IPCC states, “Limiting climate change would require substantial and sustained reductions in greenhouse gas emissions which, together with adaptation, can limit climate change risks.” (IPCC 2014a).

14 We are grateful to an anonymous reviewer for highlighting these two objections. (This referee specifically highlighted the examples of the audio engineer and the biomedical engineer.)

15 We are grateful to Dr. Andrew Stanners for discussions about research in the medical profession.

16 Carbon dioxide *equivalent* emissions.

17 For more on this idea, see Davis (1991) and (Lawlor 2016)  and (Lawlor and Morley 2016).

18 This judgement was confirmed, in conversation, by David Orr—the chair of the Institution of Civil Engineers' ethics committee (and previously president of the Institution).

19 Feedback form—quoted with permission.

20 Our discussion of engineering as an enabling activity is largely influenced by Bowen (2014).
